# A Rare Case Report of Morvan Syndrome: A Constellation of Autonomic, Central and Peripheral Nervous System Involvement

**DOI:** 10.7759/cureus.92224

**Published:** 2025-09-13

**Authors:** Alekhya K Shetty, Siddharth Gosavi, Raviraja V Acharya, Chandana Acharya

**Affiliations:** 1 Internal Medicine, Kasturba Medical College, Manipal, Manipal Academy of Higher Education, Manipal, IND

**Keywords:** morvan's syndrome, neuro-immunology, neurology, peripheral nerve disorders, rituximab

## Abstract

Morvan syndrome is an extremely rare neurological autoimmune entity that can be life-threatening for the patient if not detected early. It consists of features of autonomic, peripheral, and central nervous systems, which include fluctuating blood pressure, insomnia, delirium, cognitive decline, hyperhidrosis, and peripheral nerve hyperexcitability in the form of myokymia. It is named after the famous French physician Augustin Marie Morvan. Antibodies against voltage-gated potassium channels have been implicated in this disease. Appropriate immunosuppression and plasmapheresis are commonly used to treat this disease. We present a rare case report of Morvan syndrome from a tertiary care center in Karnataka, South India.

## Introduction

Morvan syndrome is a rare neurological disorder characterized by peripheral nerve hyperexcitability, autonomic dysfunction, and central nervous system symptoms. It is considered an autoimmune disorder. A few cases of Morvan syndrome have been linked to heavy metal exposure. It is also commonly associated with thymoma and occasionally with malignancies of the lung and prostate, suggesting a paraneoplastic etiology [1]. So far, around 60 cases of CASPR2-related Morvan syndrome have been described in French publications. In the English peer-reviewed literature, only two series are available, together comprising 43 patients. According to Orphanet (accessed January 2024), the estimated prevalence of Morvan syndrome is fewer than one case per million [1]. No prevalence estimate exists with regard to Morvan syndrome cases in India. However, fewer than 50 cases have been reported so far, with the first case reported in the year 2007 [2]. 

Morvan syndrome is associated with antibodies against voltage-gated potassium channels (VGKC). Two VGKC-related proteins have been studied in the pathogenesis of Morvan syndrome: contactin-associated protein-like 2 (CASPR2) and leucine-rich glioma-inactivated 1 (LGI1) antibodies [3]. CASPR2 is predominantly expressed in the peripheral nervous system, while LGI1 is mainly expressed in the central nervous system. Antibodies to LGI1 are associated with conditions such as limbic encephalitis, rapid eye movement (REM) sleep behavior disorders, and faciobrachial dystonic seizures. The spectrum of CASPR2 antibodies is more diverse and can be associated with conditions such as neuromyotonia, epilepsy, and pain syndromes [4].

The triad of features seen in patients with Morvan syndrome includes:

Peripheral nervous system (PNS) involvement and peripheral nerve hyperexcitability lead to continuous muscle fiber activity, resulting in twitching movements (myokymia), paresthesias or neuropathic pain, and areflexia. Central nervous system (CNS) involvement symptoms include insomnia, memory disturbances, hallucinations, confusion, or agitation [5]. Severe insomnia is a prominent feature of Morvan syndrome and may be accompanied by episodes of agitation and abnormal motor behavior [6]. Autonomic nervous system (ANS) involvement symptoms include fluctuating blood pressure, tachycardia, hyperhidrosis, and arrhythmias.

The diagnosis of Morvan syndrome is often challenging, as it necessitates the exclusion of other conditions with overlapping clinical features, such as autoimmune encephalitis, epileptic syndromes, and paraneoplastic syndromes. Morvan syndrome is primarily diagnosed based on clinical presentation along with associated electromyography changes and serum autoimmune encephalitis panel. Management primarily involves immunosuppressive therapy, and early initiation of treatment is associated with a favorable prognosis.

## Case presentation

We report the case of a 40-year-old male with no prior comorbidities, who presented to Kasturba Hospital, Manipal, with a history of paresthesias and muscle pain. The symptoms were asymmetrical and intermittent in nature, initially limited to the bilateral lower limbs, and later progressing to involve the bilateral upper limbs over a period of three months. However, the patient did not report any limb weakness and was able to carry out his daily routine activities. He also noted intermittent spontaneous twitching movements in the calf and thigh muscles, lasting for a few seconds. Additionally, the patient reported severe sweating episodes and decreased sleep for the past month and complete insomnia over the last week. Other potential predisposing factors, including a history of autoimmune disease, exposure to heavy metals or ayurvedic/herbal medications, and recent infections, were thoroughly excluded (Video 1).

**Video 1:** Fasciculations over the calf muscle.

The patient had a history of multiple hospital visits for the same complaints. He was previously admitted to the psychiatry department due to insomnia and was started on antidepressants and anxiolytics. 

Neurological examination revealed intermittent fasciculations over the bilateral calf and thigh muscles lasting for a few seconds and resolved spontaneously. The rest of the central nervous system, including higher mental function, motor system, and sensory system examination, along with the systemic examination, was unremarkable.

On evaluation, routine laboratory parameters revealed hyponatremia. The response to intravenous fluids was poor. Hence a trial of selective vasopressin antagonist (tolvaptan) was initiated. The sodium levels initially measured 114 mmol/L and rose to 120 mmol/L over a period of 24 hours with 15 mg tolvaptan. However, it remained between 120 and 125 mmol/L until the initiation of immunosuppressive agents, after which it improved to 135 mmol/L. Creatine phosphokinase (CPK) levels were elevated. Brain imaging (MRI brain) was normal. Other laboratory parameters were within normal limits. A paraneoplastic workup, which included CD19, CD20, CEA, CA19-9, and AFP, was negative.

Table 1 depicts the laboratory parameters.

**Table 1:** Laboratory parameters. CPK: Creatine phosphokinase, TSH: Thyroid-stimulating hormone

Test	Result	Reference range
Sodium (serum)	114.2 mmol/L	135-145 mmol/L
CPK (serum)	711.0 U/L	55-170 U/L
Vitamin b12	>2000 pg/mL	200-900pg/mL
TSH	3.320 microIU/mL	0.4-4.0 microIU/mL
Antinuclear Antibody profile	Negative	
Absolute CD45 + Lymphocyte count	1435.47 /microL	1000-4800 cells/microL
Absolute CD19+CD20+ count	374.98 /microL	50-600 cells/microL

**Figure 1:**
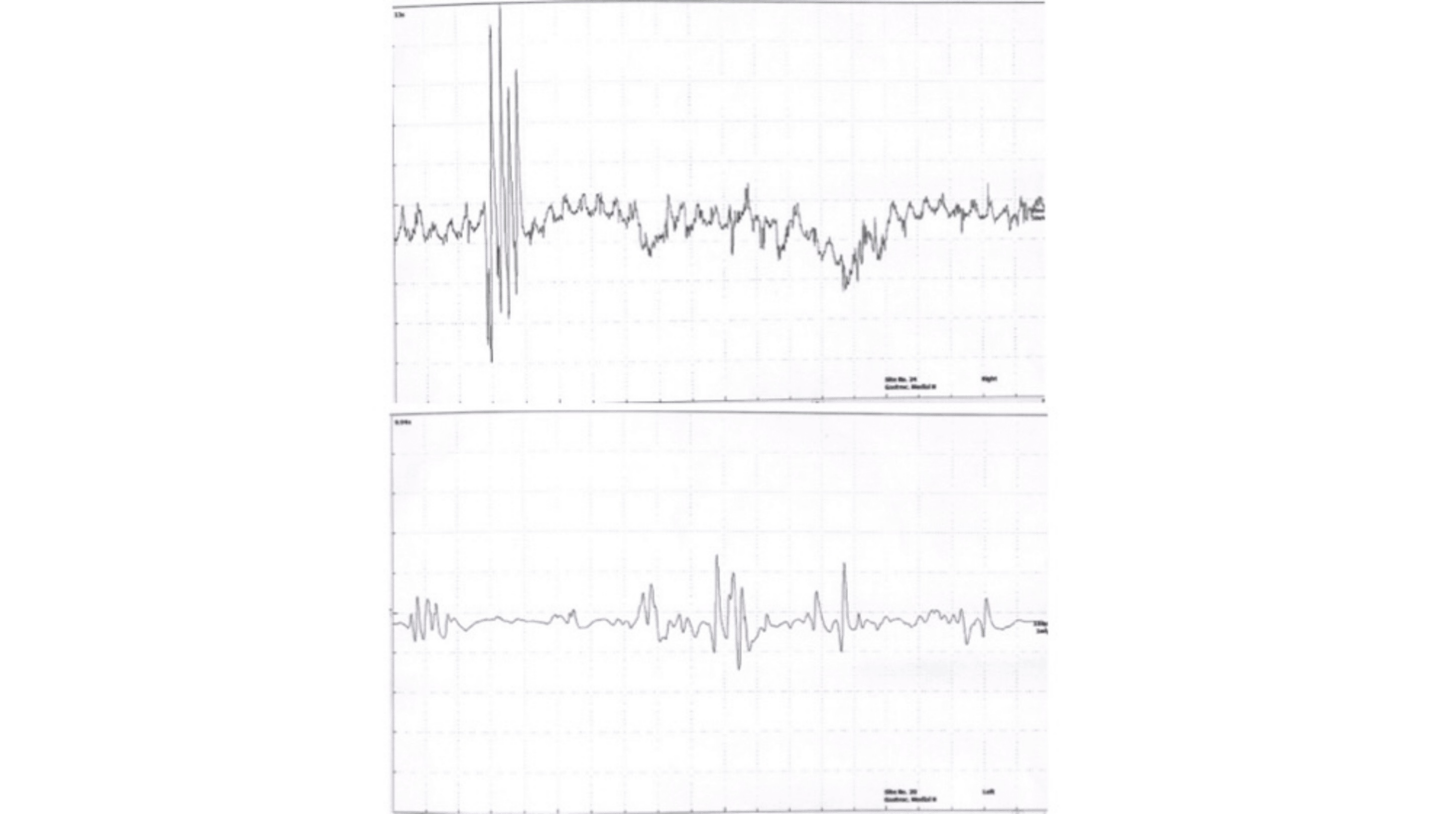
Electromyography (EMG) of right and left gastrocnemius muscles.

Electromyography (EMG) was performed due to the suspicion of a neurological or neuromuscular disorder. Needle EMG sampling, as shown in Figure 1, demonstrates irregular high-frequency bursts in the form of doublets, triplets, and fasciculations in the bilateral gastrocnemius muscles. In the clinical context of myokymia, these findings are suggestive of peripheral nerve hyperexcitability, consistent with neuromyotonia.

As the EMG revealed fasciculations, an autoimmune encephalitis panel was ordered to confirm the diagnosis. The serum autoimmune encephalitis panel tested positive for antibodies against CASPR2 (contactin-associated protein-like 2), a protein associated with voltage-gated potassium channels (VGKC).

Table 2 shows the patient's serum autoimmune encephalitis mosaic report.

**Table 2:** Serum autoimmune encephalitis panel report. NMDA: N-methyl-D-aspartate, VGKC: Voltage-gated potassium channels, GABA-B: Gamma-aminobutyric acid type B receptor

Antibodies against	Report
Glutamate receptor, NMDA	Negative
Glutamate receptor, AMPA1	Negative
Glutamate receptor, AMPA2	Negative
CASPR (contactin associated protein 2/ VGKC associated)	Strongly positive
LGI-1 (Leucine rich glioma-inactivatedprotein 1/ VGKC associated)	Negative
GABA B receptor	Negative

Method of testing: Indirect immunofluorescence assay on transfected cell lines, used for the qualitative determination of human antibodies against glutamate and VGKC receptors in serum.

Hence, based on the clinical history, examination findings, EMG findings, and the serum autoimmune panel, a diagnosis of Morvan syndrome was made. Our patient received intravenous pulse steroids (methylprednisolone 1 gram daily) for five days, followed by the first dose of intravenous Rituximab, 1 gram. The second dose of rituximab was administered three weeks later. The patient was continued on oral corticosteroids, which were tapered over 1-2 months. For symptomatic relief, a benzodiazepine (clonazepam) and a GABA agonist (gabapentin) were prescribed briefly, although their efficacy in Morvan’s syndrome remains uncertain. The next dose of rituximab (second dose of the first cycle) was given after three weeks. Further cycles of rituximab therapy have been planned after a duration of 3-6 months. The hyponatremia started to improve immediately after the initial pulse therapy. Following the first cycle of rituximab therapy, the patient reported marked symptomatic improvement, with resolution of insomnia and sweating episodes and a relative reduction in fasciculations, though paresthesias persisted.

## Discussion

CASPR2 autoimmune encephalitis is an extremely rare condition. Voltage-gated potassium channel (VGKC) antibodies are known to be associated with three main conditions: neuromyotonia, Morvan’s syndrome, and limbic encephalitis [7]. Several proteins are associated with these channels, including LGI1, CASPR2, Contactin-2, DPPX, ADAM22, and ADAM23. Contactin-associated protein-like 2 (CASPR2) plays a critical role in the functioning of VGKCs. It binds to Contactin-2 and helps cluster Kv1 potassium channels in the juxtaparanodal region, which is essential for proper myelination of axons [2]. CASPR2 is predominantly expressed in the peripheral nervous system (PNS). Neuromyotonia, a manifestation of peripheral nerve hyperexcitability, is a relatively specific feature of CASPR2-associated conditions, highlighting its expression mainly in the PNS [8].

The diagnostic approach to Morvan’s syndrome is multidisciplinary. It is crucial to rule out other conditions such as autoimmune encephalitis, paraneoplastic syndromes, and heavy metal toxicity (e.g., mercury), which may present with overlapping clinical features [9]. Morvan syndrome can resemble pheochromocytoma due to its autonomic hyperactivity features, which mimic the catecholamine surge typically seen in pheochromocytoma [10]. Autoimmune encephalitis was considered unlikely, as there were other features of peripheral nerve excitability and the MRI of the brain was normal. The serum autoimmune encephalitis panel was also negative for antibodies specific to autoimmune encephalitis, including N-methyl-D-aspartate (NMDA) antibodies. Neuroimaging studies (MRI) are often normal in patients with Morvan’s syndrome. However, electroencephalogram (EEG) may show diffuse slow waves and a characteristic REM sleep pattern with absent non-REM sleep stages [11]. Electromyography (EMG) is essential for diagnosing neuromyotonia in Morvan’s syndrome, characterized by fasciculations, generalized multiplets, and neuromyotonic discharges, hallmarks of peripheral nerve hyperexcitability [12]. Therefore, diagnosis can be confirmed by identifying neuromuscular hyperexcitability on EMG, the presence of serum VGKC autoantibodies, and sleep-wave abnormalities on polysomnography.

Our patient was a 40-year-old male with no prior comorbidities. The clinical spectrum of this disease is notably diverse. In our case, the patient presented with features of neuromyotonia, paresthesias, autonomic dysfunction (in the form of hyperhidrosis and severe insomnia), and hyponatremia secondary to syndrome of inappropriate antidiuretic hormone secretion (SIADH) [13]. While LGI1 antibodies are more commonly associated with neuropsychiatric features and hyponatremia, CASPR2 antibodies are typically linked to peripheral nerve hyperexcitability. Demirbaş et al. reported that isolated CASPR2 antibody positivity with concurrent hyponatremia is uncommon, and in their case, hyponatremia persisted despite fluid restriction and other interventions. Similarly, in our patient, only the CASPR2 antibody was detected, and he also developed persistent hyponatremia. The hyponatremia in our case did not show a response to intravenous fluids and showed only a modest response to tolvaptan therapy. It persisted throughout the clinical course until the final diagnosis and improved to the normal range only after initiation of IV steroids, highlighting a rare yet important association of Morvan syndrome with CASPR2 positivity and hyponatremia [14]. 

This case report contributes to the growing awareness of Morvan’s syndrome. Though rare, it should be considered as a differential diagnosis in patients presenting with a wide range of symptoms involving the central nervous system, peripheral nervous system, and autonomic dysfunction. In developing countries like India, diagnostic challenges may lead to underdiagnosis or misdiagnosis. Importantly, this condition responds well to immunosuppressive therapy when initiated early, emphasizing the need for timely diagnosis to improve outcomes.

The primary goal of treatment in Morvan’s syndrome is immunosuppression. In our patient, immunotherapy was initiated early in the disease course. He received pulse steroid therapy and Rituximab. The condition typically responds to intravenous immunoglobulin (IVIg), plasma exchange, and immunosuppressive agents [15]. Therapeutic plasma exchange may be used in the acute phase to reduce circulating antibody levels, particularly when there is an inadequate response to corticosteroids. Long-term management relies on immunosuppressive therapy [16]. Some case reports suggest that symptoms refractory to plasma exchange or corticosteroids may respond to steroid-sparing agents such as azathioprine, rituximab, or even lacosamide [1]. Rituximab has shown a good response in autoimmune encephalitis, particularly in NMDA receptor encephalitis, and has also demonstrated efficacy in LGI1 and CASPR2-associated encephalitis, though data are more limited compared to NMDA-associated cases [17].

## Conclusions

Early treatment with immunosuppression significantly contributes to a favorable prognosis in patients with Morvan syndrome. Prompt evaluation for autoimmune encephalitis and potential paraneoplastic etiology is crucial. As observed in many cases, the condition may initially be misdiagnosed as peripheral neuropathy, depression, or primary insomnia. Therefore, it is imperative for physicians to recognize the constellation of symptoms and clinical signs early in the disease course. Physicians should also be familiar with the clinical sign of myokymia, characterized by continuous rippling fasciculations, as it is a key feature of peripheral nerve hyperexcitability and can aid in narrowing the differential diagnosis.
